# Evaluating Avoidable Transfers of Pediatric Forearm Fractures to the Emergency Department

**DOI:** 10.7759/cureus.85103

**Published:** 2025-05-30

**Authors:** Adrianna Kowblansky, Andrew Fealy, Sarah Dance, Adam Mansour, Kevin Cleary, Sean A Tabaie

**Affiliations:** 1 Orthopedic Surgery, George Washington University School of Medicine and Health Sciences, Washington DC, USA; 2 Orthopedic Surgery, Children’s National Hospital, Washington DC, USA; 3 Orthopedic Surgery, Nationwide Children’s Hospital, Columbus, USA; 4 Bioengineering, Children’s National Hospital, Sheikh Zayed Institute for Pediatric Surgical Innovation, Washington DC, USA

**Keywords:** forearm fracture management, pediatric emergency department (ped), pediatric forearm fracture, pediatric fractures, pediatric orthopedic

## Abstract

Background

Forearm fractures represent approximately 20% of pediatric fractures and are commonly managed in emergency departments (EDs). While minimally displaced fractures require immobilization, displaced fractures necessitate reduction. Many non-pediatric facilities lack the resources to manage these fractures, leading to frequent transfers to pediatric emergency departments (PEDs). This study aims to evaluate the rate of avoidable transfers of pediatric forearm fractures to a PED and identify risk factors contributing to unnecessary transfers.

Methods

A retrospective cross-sectional study was conducted at a single tertiary pediatric hospital from July 1, 2022, to June 30, 2023. Patients aged 0-17 years treated for forearm fractures were identified using ICD-10 codes. Exclusion criteria included patients not transferred, incomplete treatment data, or missing initial radiographs. Collected variables encompassed demographics, fracture characteristics, transfer details, and treatments performed. Statistical analyses included chi-square tests for categorical variables and Mann-Whitney U tests for continuous variables, with significance set at p < 0.05.

Results

Out of 445 patients identified, 161 met the inclusion criteria. The mean age was 8.0 years (SD 3.9); 70.8% were male, and 31.7% were African Americans. Avoidable transfers, defined as patients who did not require reduction or surgical intervention, accounted for 38 cases (23.6%). Non-displaced fractures were significantly associated with avoidable transfers (p < 0.001). Race was also significant, with higher rates of avoidable transfer among African American patients (42.1%) and patients of other races (47.4%) compared to Caucasian patients (10.5%) (p = 0.006). There was a statistically significant association between the source of appropriate and avoidable transfers (p = 0.012), with cases originating from clinics (n = 7), outside hospitals (n = 24), and urgent care centers (n = 7). Multivariate logistic regression identified younger age as the only significant factor associated with avoidable transfer (p = 0.047, OR: 0.74, CI: 0.5-0.98).

Conclusions

A significant proportion of pediatric forearm fracture transfers to the PED were avoidable, primarily due to patients not requiring reduction or surgical intervention. Younger age was a significant factor associated with unnecessary transfers. Enhancing education on pediatric fracture management and establishing clear guidelines may reduce unnecessary transfers, optimize resource utilization, and alleviate burdens on specialized centers.

## Introduction

Fractures are among the most common reasons for pediatric emergency department (PED) visits in the United States, representing a significant public health concern [1,2]. The incidence of pediatric fractures varies widely, reported between 12 and 36 per 1,000 children annually [3]. Forearm fractures account for approximately 20% of all pediatric fractures [1]. These fractures commonly result from trauma, such as falls onto an outstretched hand, especially in active children [4]. 

Pediatric forearm fractures are classified based on bone alignment into non-displaced, minimally displaced, and displaced fractures [5,6]. Non-displaced and minimally displaced fractures typically require only immobilization with casting or splinting, whereas displaced fractures often necessitate reduction to realign the bones properly prior to immobilization [5,7]. In pediatric patients, ketamine sedation is commonly used during fracture reduction to minimize pain and psychological trauma [8,9]. However, many adult-oriented emergency departments (EDs) or outpatient centers lack pediatric-specific sedation protocols and equipment necessary for safe fracture reduction in children [10]. Consequently, these patients are often transferred to PED for further management, increasing the burden on pediatric emergency and orthopedic services and leading to inefficient use of healthcare resources [11,12]. 

The financial implications of ED visits for pediatric forearm fractures are significant. A 2019 study by Godfrey et al. discovered a median cost of $1390 for closed treatment without manipulation in the ED with closed reduction adding an additional $894 in treatment costs [8]. Furthermore, there is an inconvenience factor that is placed on the family that must drive to an additional facility for casting or splinting, and later follow-up care. Given these factors, it is crucial to assess the extent of unnecessary referrals of pediatric fracture patients to PED for management. In this study, an avoidable transfer is defined as a patient transferred to a tertiary PED who does not require reduction or surgical intervention. The purpose of the present study is to evaluate the rate of avoidable transfers of pediatric forearm fractures in a tertiary PED and to identify risk factors contributing to these unnecessary transfers.

## Materials and methods

Patient selection 

The present study was an institutional review board-approved, retrospective cross-sectional chart review of pediatric patients (aged 0 to 17 years) who presented at a tertiary PED between July 2022 and June 2023. International Classification of Diseases - 10th Revision (ICD-10) codes S52.0, S52.1, S52.2, S52.3, S52.4, S52.5, S52.6, and S52.9 were used to identify patients with a forearm fracture involving either the radius, ulna, or both. Patients who were not transferred to the PED from a different primary site, patients who had incomplete treatment data such as poor documentation on treatment at tertiary PED, and patients who were missing primary site radiographs were excluded. Patients were stratified as appropriate or avoidable transfers. Appropriate transfers included those who required a reduction prior to casting/splinting or operative intervention. Avoidable transfers included those who received casting/splinting without reduction or no intervention at the tertiary site.

Baseline characteristics 

Demographics included age, sex, race/ethnicity, and date of injury. Injury data collected included age of fracture at ER presentation, laterality of injury, location of ulnar and radial fracture(s), fracture displacement, if it was an open fracture, if it was a non-vascular injury (NVI), if it was a refracture, if there was Monteggia or Galleazi joint instability, and if there were additional fractures not in the forearm. Transfer data collected included whether the patient was transferred/referred, the kind of facility that transferred/referred the patient to the ER, and the reason for transfer/referral. Treatment data included whether a reduction was needed and if the injury required splinting, casting, open surgery, or no further treatment. 

Statistical analysis 

Statistical analyses were performed using R version 4.0.6. Univariate analyses were conducted, with categorical variables compared using chi-square or Fisher's exact tests, and continuous variables analyzed using the Mann-Whitney U test due to non-normal distribution. A p-value of less than 0.05 was considered statistically significant. Variables found to be significant in univariate analysis were included in a multivariate logistic regression model. Results were reported with 95% confidence intervals.

## Results

Patient demographics 

A total of 445 pediatric patients who were seen at the PED for a forearm fracture were initially identified, 161 of them met the inclusion criteria. Many patients were excluded because they presented to the PED without being referred from a primary facility. The mean age of the cohort was 8.0 (QR: 5.0 11.0). One hundred fourteen patients were male (70.8%) and 47 (29.2%) patients were female. Fifty-one (31.7%) patients were Caucasian, 51 (31.7%) patients were African American, and 59 (36.6%) patients were defined as “other race,” which included Asian/Pacific Islander, American Indian, another race that was not a survey option, mixed race, and declined to answer. Additional demographics are detailed in Table 1.

**Table 1:** Demographics of all transferred pediatric forearm fractures *Statistical significance (p<0.05)

Characteristics	Total Transferred Patients (N = 161)	Appropriate Transfers (N = 123)	Avoidable Transfers (N = 38)	P-Value
Age (years), Median (IQR)	8.0 (5.0 11.0)	8.0 (6.0 11.5)	6.0 (4.2 9.0)	0.019*
Sex, n (%)				0.11
Male	114 (70.8%)	91 (74.0%)	23 (60.5%)	
Female	47 (29.2%)	32 (26.0%)	15 (39.5%)	
Race/Ethnicity, n (%)				0.006*
Caucasian	51 (31.7%)	47 (38.2%)	4 (10.5%)	
African American	51 (31.7%)	35 (28.5%)	16 (42.1%)	
Other Race	59 (36.6%)	41 (33.3%)	18 (47.4%)	

Injury data 

**Figure 1:**
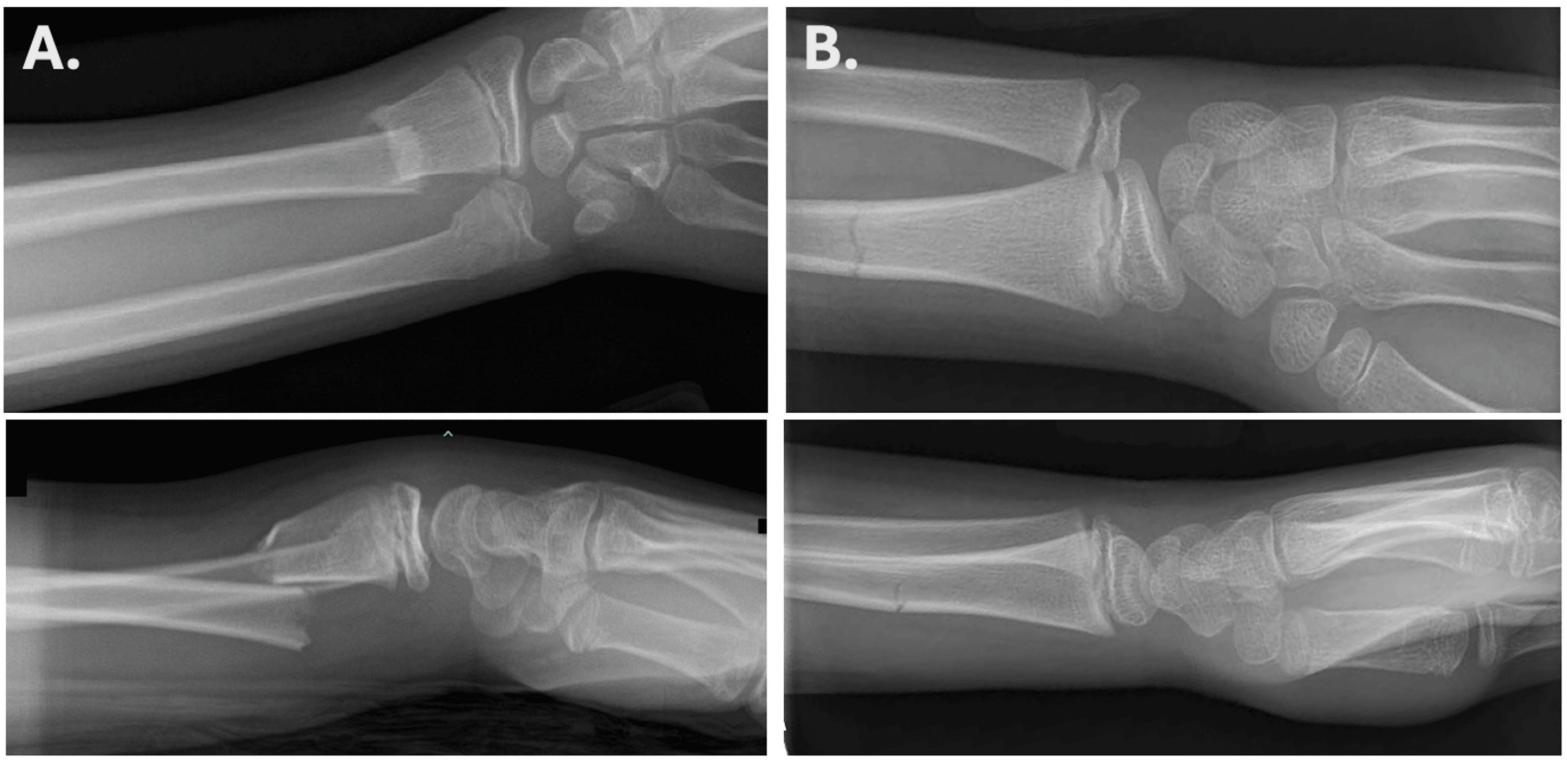
(A) Displaced fracture; (B) Non-displaced fracture

The mean time from initial injury to PED presentation was 0 days (IQR: 0-1) (Table 2). One hundred (62.1%) patients had ulnar fractures: 11 (6.8%) were physeal, 59 (36.6%) were distal, 21 (13.0%) were midshaft, and nine (5.6%) were proximal. One hundred fifty-two (94.4%) patients had radius fractures: 20 (12.5%) were physeal, 97 (60.6%) were distal, 25 (15.6%) were midshaft, and 10 (6.2%) were proximal. Ninety-two (57.1%) patients had both an ulna and radius fracture. Thirty-two (19.9%) of fractures were non-displaced, and 129 (80.1%) were displaced fractures (Figure 1). Five (3.1%) patients had an open fracture, and two (1.2%) patients had a vascular injury. No patients were presenting due to a refracture. Six (3.7%) patients had joint instability. Additional fractures not localized to the forearm were seen in four (2.5%) patients. 

**Table 2:** Fracture injury characteristics of transferred patients *Statistical significance (p<0.05) NA, not applicable

Characteristics	Transferred Patients (N = 161)	Appropriate Transfers (N = 123)	Avoidable Transfers (N = 38)	P-Value
Time to ER Presentation (Days), Mean (IQR)	0 (0-1)	0.0 (0-1)	1.5 (0.2-4.0)	<0.001*
Ulnar Fracture, n (%)				0.355
Physeal	11 (6.8%)	9 (7.3%)	2 (5.3%)	
Distal	59 (36.6%)	48 (39%.0)	11 (28.9%)	
Midshaft	21 (13.0%)	18 (14.6%)	3 (7.9%)	
Proximal	9 (5.6%)	6 (4.9%)	3 (7.9%)	
Radius Fracture, n (%)				0.159
Physeal	20 (12.5%)	18 (14.6%0	2 (5.4%)	
Distal	97 (60.6%)	72 (58.5%)	25 (67.6%)	
Midshaft	25 (15.6%)	22 (17.9%)	3 (8.1%)	
Proximal	10 (6.2%)	6 (4.9%)	4 (10.8%)	
Displacement, n (%)				<0.001*
Non-displaced	32 (19.9%)	1 (0.8%)	31 (81.6%)	
Displaced	129 (80.1%)	122 (99.2%)	7 (18.4%)	
Open Fracture, n (%)	5 (3.1%)	NA	NA	NA
Vascular Injury, n (%)	2 (1.2%)	NA	NA	NA
Joint Instability, n (%)	6 (3.7%)	NA	NA	NA
Additional Fractures, n (%)	4 (2.5%)	NA	NA	NA

Transfer and treatment data 

Among the 161 patients who were transferred to the tertiary PED, 12 (7.5%) were referred from a clinic, 39 (24.2%) were referred from urgent care, and 110 (68.3%) were referred from an outside hospital. Seventy-eight of these patients (48.8%) were referred due to a “failure to treat the fracture” at the primary site. The remaining 83 patients (51.6%) did not report a reason for their referral. Of note, these reasons for referral were self-reported by either the patient or the patient’s parent/guardian upon intake at the PED. All patients included in this study presented at the tertiary PED from which an orthopedic consult was requested.

Once at the tertiary PED, 38 patients (23.6%) did not require a reduction. Of these, 17 (10.6%) of these patients were splinted, 19 (11.8%) were casted, and two patients (1.2%) received no intervention once transferred. One hundred twenty-three (76.4%) patients who were transferred required reduction or surgical intervention; four patients (2.5%) underwent closed reduction and splinting, 98 patients (60.9%) underwent closed reduction and casting, and 21 patients (13.0%) went to surgery for open reduction.

Thirty-eight patients (23.6%) were avoidable transfers according to this definition. Univariate analysis revealed that avoidable transfer was statistically significantly related to younger age (p = 0.019), race (p = 0.006), fracture age (p < 0.001), and from the type of facility the patient was transferred/referred from (p = 0.012) (Table 3). Multivariate logistic regression was also performed from which only age was significantly associated with increased odds of avoidable transfer (p = 0.047) (Table 4). 

**Table 3:** Transfer and treatment characteristics of injuries in transferred patients *Statistical significance (p<0.05) PED, pediatric emergency department; NA, not applicable

Characteristics	Transferred Patients (N = 161)	Appropriate Transfers (N = 123)	Avoidable Transfers (N = 38)	P-Value
Referral Source, n (%)				0.012*
Clinic	12 (7.5%)	5 (4.1%)	7 (18.4%)	
Urgent Care	39 (24.2%)	32 (26.0%)	7 (18.4%)	
Outside Hospital	110 (68.3%)	86 (69.9%)	24 (63.2%)	
Reason for Transfer, n (%)				<0.001*
Failure to Treat	78 (48.8%)	65 (53.3%)	13 (34.2%)	
Not Provided	83 (51.6%)	57 (46.3%)	25 (65.8%)	
Treatment at Tertiary PED, n (%)				NA
No Reduction and Splint	17 (10.6%)	0 (0%)	17 (10.6%)	
No Reduction and Cast	19 (11.8%)	0 (0%)	19 (11.8%)	
Reduction and Splint	4 (2.5%)	4 (2.5%)	0 (0%)	
Reduction and Cast	98 (60.9%)	98 (60.9%)	0 (0%)	
Surgery	21 (13.0%)	21 (13.0%)	0 (0%)	
No Intervention	2 (1.2%)	0 (0%)	2 (1.2%)	

**Table 4:** Multivariate analysis *Statistical significance aOR, adjusted odds ratio

Variables	Level	aOR (95% Cl)	P-Value
Age (year)	Per 1 Unit Increase	0.74 (0.5-0.98)	0.047*
Race/Ethnicity			
	Caucasian	Reference	
	African American	16.28 (0.96-1205.91)	0.110
	All Other	21.25 (1.12-2093.47)	0.096
Fracture Age (Days)		1.43 (1.07-2.48)	0.160
Fracture Type			
	Non-displaced	Reference	
	Displaced	0.00 (0.00-0.01)	<0.001*
Transfer Source			
	Clinic	Reference	
	Urgent Care	1.16 (0.02-156.59)	0.949
	Outside Hospital	1.42 (0.03-179.17)	0.881
Reason for Transfer			
	Not Given	Reference	
	Unable to Treat	0.81 (0.11-5.58)	0.824

## Discussion

The present study evaluated the rate of avoidable transfers of pediatric forearm fractures to a tertiary PED. Additionally, it identified risk factors that may contribute to these unnecessary transfers. The findings suggested that 23.6% of patients were avoidable transfers, meaning they did not require reduction or surgical intervention at the tertiary site. The analysis highlighted several risk factors for avoidable transfer, most notably younger age, race, and the source of referral.

In the United States, there are over 23 million annual PED visits [13,14]. Nearly 90% of these visits occur at non-pediatric hospitals, and a substantial percentage of patients are discharged without any intervention [13,14]. In the current study, 38 patients were considered avoidable transfers, as 17 were splinted without reduction, 19 were casted without reduction, and two received no treatment at the tertiary site. This suggests that nearly one-quarter of the transfers could have been avoided, with patients potentially adequately treated with immobilization at the referring site. Although substantial, this rate is lower than other reports in the literature indicating higher rates of unnecessary transfers. For instance, a 2022 study by Scheier et al. found that 49% of pediatric distal forearm fractures were referred to the PED from outside facilities [15]. Similarly, Godfrey et al. noted that nearly 81.6% of pediatric distal forearm fractures at a large academic children's hospital did not require any reduction [8]. A possible explanation for this discrepancy may be differences in provider comfort levels and training in managing pediatric orthopedic complaints, two factors that were unable to be analyzed from the present study's data set.

As expected, the majority of displaced pediatric forearm fractures transferred to the PED (122 of 129, 94.6%) required additional treatment. The 38 (23.6%) fractures that were transferred, 31 non-displaced and seven displaced, but did not require interventions beyond splinting or casting are of particular concern. It is understandable that fracture displacement status was significantly correlated with avoidable referral (p < 0.001). The substantial proportion of avoidably transferred patients underscores the need for improved triage protocols and enhanced communication between referring and receiving centers. Such improvements could include implementing a system whereby the referring facility initiates a phone call with the receiving PED or orthopedic team to discuss the patient and review imaging. The goal of such a system is to enhance communication and appropriate referral practice. Avoidable referrals to the ED are a deep-seated issue in U.S. healthcare [16], often leading to over-treatment. A recent study found that up to 20% of medical treatments and referrals were unnecessary [17]. By implementing standardized practice guidelines and improved triage protocols at smaller community hospitals and urgent care centers, many unnecessary referrals, and the associated waste of hospital resources, can likely be reduced. 

Several variables were associated with avoidable transfer, including younger patient age (p = 0.019), race (p = 0.006), time from injury to ER presentation (p < 0.01), and source of transfer (p = 0.012). Specifically, for each one-year increase in age, patients were 26% less likely to be avoidably transferred. This finding aligns with previous studies highlighting the over-transfer of younger pediatric patients to EDs for various conditions, such as asthma exacerbations [13,18]. A possible explanation for increased transfers among younger patients in the current cohort is the desire for more cautious management due to open growth plates. 

Although race was significantly associated with avoidable transfer on univariate analysis (p = 0.006), multivariate analysis did not find any single racial group to be significant (African American race: p = 0.11; other races: p = 0.096). This contrasts with previous studies that found Hispanic or African American patients were more likely to experience avoidable transfers [13,18,19]. Therefore, it is plausible that with a larger cohort, an association between race and avoidable transfer might become evident in multivariate analysis. As such, the impact of race on ED transfers warrants further investigation. 

The source of transfer, whether from a clinic, urgent care center, or outside hospital, was also statistically significant in the univariate analysis. Nearly half of the patients (48.8%) were transferred due to self-reported inability to treat the fracture at the initial site, while 51.2% had no self-reported reason for transfer. A 2021 narrative review by Caruso et al. concluded that there is no consensus on the management of pediatric forearm fractures [20], instead providers must rely on management principles as evidenced in the medical literature. This lack of standardized management guidelines may contribute to uncertainty among providers regarding when transfers are necessary. Enhancing education and training for providers at primary care and urgent care centers could help reduce the number of avoidable transfers. Alternatively, implementing the use of telemedicine to consult a pediatric orthopedic surgeon could provide a valuable second opinion when the decision to refer the patient is uncertain. This could also enable direct referral to an outpatient pediatric orthopedic surgeon rather than the PED for non-emergent cases. A 2023 pilot study by Mowrer et al. investigated the use of a structured phone survey to guide pediatric orthopedic transfers from smaller facilities to a Level 1 pediatric trauma center, in alignment with American Academy of Pediatrics (AAP) guidelines [21]. Despite this intervention, the study found that 64% of transfers did not meet the criteria and were potentially avoidable. These findings underscore that even with standardized communication protocols, a substantial number of unnecessary transfers may still occur.

While the present study provides valuable insights into the rates and risk factors of avoidable transfers of pediatric forearm fractures, several limitations must be acknowledged. The study's sample size, although reasonable, may not fully capture all potential variables influencing transfer decisions, particularly due to the small numbers within certain racial and ethnic subgroups. Additionally, since the present study's data was from the tertiary pediatric site, it lacked detailed information about the resources and capabilities of the transferring facilities, which could influence transfer decisions. Furthermore, the current study could not identify patients who were referred to the tertiary PED but did not present, potentially leading to selection bias. These factors may affect the generalizability of the findings and limit the understanding of the rationale behind deemed avoidable transfers.

Future research should focus on prospective studies evaluating the implementation of standardized triage protocols and decision-making tools for pediatric forearm fractures, as well as assessing the long-term outcomes of both appropriately and avoidably transferred patients. Additionally, larger cohort studies stratified by insurance status, geographic region, and other social determinants of health may help elucidate their influence on the transfer process. 

## Conclusions

In conclusion, nearly one-quarter (23.6%) of pediatric patients with forearm fractures were transferred to a tertiary PED, a transfer that could potentially have been avoided through treatment at a lower-echelon facility. Younger age was the most significant predictive factor associated with avoidable transfers in the present study. By identifying gaps in care through similar studies, improvements in pediatric orthopedic management can be achieved at primary sites, unnecessary healthcare costs can be reduced, and resource allocation can be optimized.
